# The complete chloroplast genome sequence of *Butea monosperma* (Fabaceae)

**DOI:** 10.1080/23802359.2020.1811173

**Published:** 2020-08-28

**Authors:** Dan Wei, Xiaogang Ding, Hangyong Zhu, Zhongrui Zhang, Haiyan Yang, Renchao Zhou, Seping Dai, Geng Zhang

**Affiliations:** a Guangdong Academy of Forestry/Guangdong Provincial Key Laboratory of Silviculture, Protection and Utilization, Guangzhou, China; b State Key Laboratory of Biocontrol and Guangdong Key Laboratory of Plant Resources, School of Life Sciences, Sun Yat-sen University, Guangzhou, China; c Guangzhou Institute of Forestry and Landscape Architecture, Guangzhou, China

**Keywords:** *Butea monosperma*, medicine, complete cp genome;automated assembly

## Abstract

*Butea monosperma*, an importantmedicinal plantin Fabaceae, is mainly distributed in southern Asia. In this study, we reported the complete chloroplast (cp) genome of *B. monosperma* assembled with Illumina sequencing data. The whole cp genome of this species is 151,925 bp in length, consisting of two inverted repeat regions (IR, 25,083 bp), one large single-copy region (LSC, 83,541 bp), and one small single-copy region (SSC, 18,218 bp).A total of 128 genes were annotated for the chloroplast genome, including 83 protein-coding genes, 37 tRNAs and 8 rRNAs. Phylogenetic analysis indicated that *B. monosperma* was closely related to the genus *Lespedeza.*

*Butea monosperma*, belonging to the family Fabaceae, is a valuable medicinal plant.It is widely used in the treatment of various ailments including abdominal tumors and possesses anti-estrogenic activity (Karia et al. 2018). It also has antibacterial and antidiarrheal activity and could be applied in eradicating water borne diseases (Sharma et al. 2019). The flowers of *B. monosperma* are usually used as a natural fabric dye material for the yellow color, especially in silk industry. In this study, we reported its complete chloroplast genome sequence, which may be helpful for further genetic research of this species.

The sample of *B. monosperma* was collected from Guangzhou Tree Park, Guangzhou, China. The specimen was stored at the herbarium of Guangdong Academy of Forestry (specimen NO. ZK20200153). The genomic DNA of *B. monosperma* was extracted from fresh leaves with a Plant Genomic DNA Extraction kit (Jierui Biotech, Guangzhou, China) and sequenced using the Illumina Novaseq platform (Illumina, San Diego, CA). The cp genome was assembled by the GetOrganelle (Jin et al. 2018) software and then annotated with the Geseq (Tillich et al. 2017) online software with further manual correction. Finally, a complete chloroplast genome of *B. monosperma* was obtained and submitted to GenBank with the accession number MT240945.

The complete cp genome of *B. monosperma* is 151,925 bp in length, containing a large single-copy (LSC) region of 83,541 bp, a small single-copy (SSC) region of 18,218 bp and two inverted repeat (IR) regions of 25,083 bp. The whole cp genome contains a total of 128 genes, including 83 protein-coding genes, 37 tRNA genes, and 8 rRNA genes. The overall GC content of the genome is 35.25%, with that of the LSC, SSC and IR regions being 54.99%, 11.99%, and 33.02%, respectively.

To investigate its phylogenetic position, complete cp genomes of 14 other Fabaceae species were aligned with *B. monosperma* using MAFFT (Katoh & Standley 2013). A maximum likelihood analysis was performed by RAxML (Stamatakis 2014) with 1000 bootstrap replicates with *Sophora alopecuroides* (Genbank Accession NC_036102) as an outgroup. The phylogenetic tree showed that *B. monosperma*was closely related to the genus *Lespedeza* (Figure 1).

**Figure 1.**
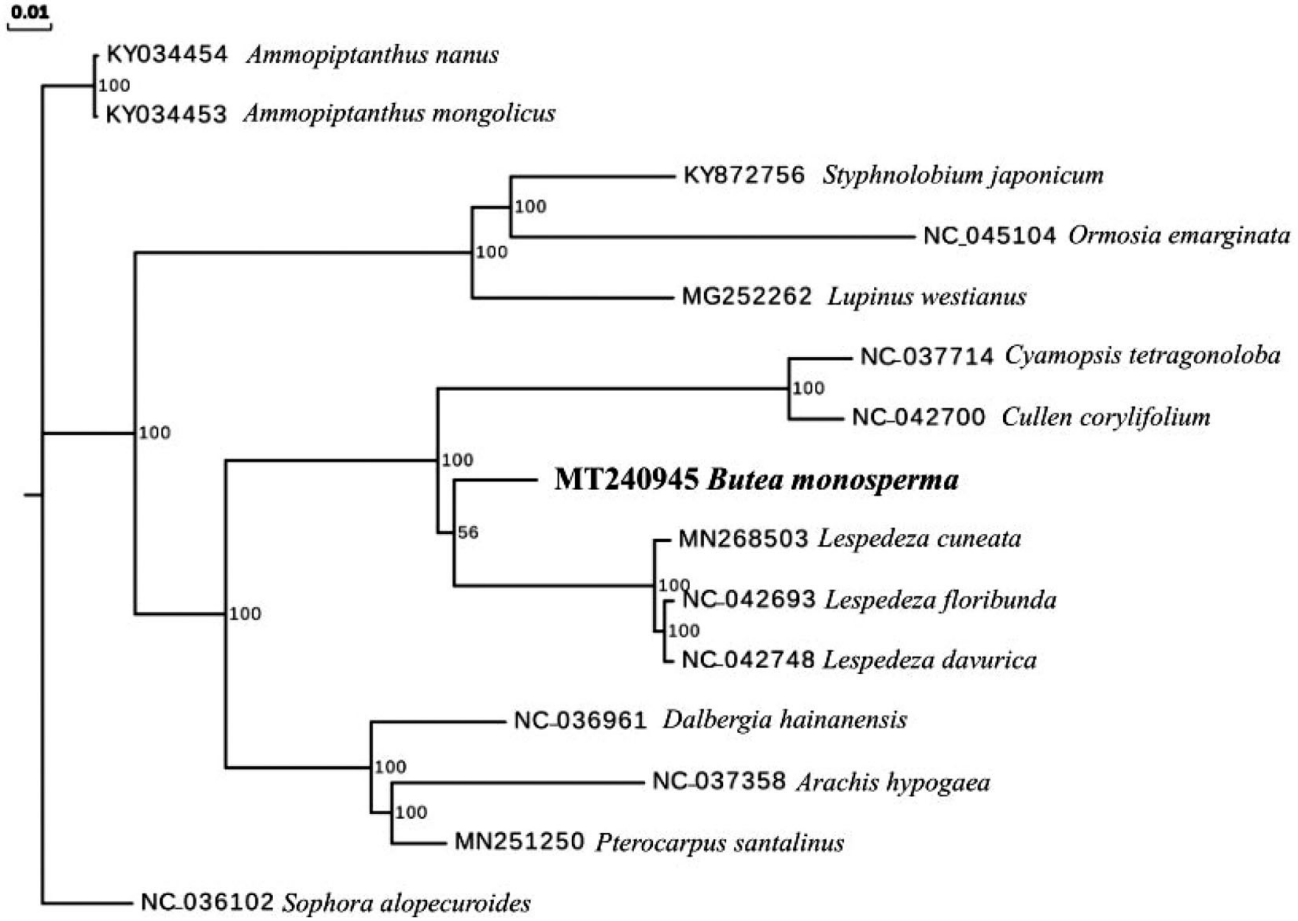
Maximum likelihood tree of 15 species in Fabaceae (including *B. monosperma*) based on their chloroplast genome sequences. Bootstrap support values (based on 1000 replicates) are shown next to the nodes.

## Data Availability

The data that support the findings of this study are available from the corresponding author, Geng Zhang, upon reasonable request. https://www.ncbi.nlm.nih.gov/search/all/?term=MT240945
